# An empirical model that uses light attenuation and plant nitrogen status to predict within-canopy nitrogen distribution and upscale photosynthesis from leaf to whole canopy

**DOI:** 10.1093/aobpla/plv116

**Published:** 2015-10-03

**Authors:** Gaëtan Louarn, Ela Frak, Serge Zaka, Jorge Prieto, Eric Lebon

**Affiliations:** 1 INRA UR4 URP3F, BP6, F86600 Lusignan, France; 2 INTA EEA Mendoza, San Martín 3853, Luján de Cuyo (5507), Mendoza, Argentina; 3 INRA, UMR 759, LEPSE, 2 place Viala, F34060 Montpellier, France

**Keywords:** Light, *Medicago sativa*, nitrogen distribution, nitrogen nutrition index, photosynthesis, transpiration, upscaling, within-canopy variability

## Abstract

Modelling the spatial and temporal distribution of leaf nitrogen (N) is central to specify photosynthetic parameters and simulate canopy photosynthesis. Leaf photosynthetic parameters depend on both local light availability and whole-plant N status. The interaction between these two levels of integration has generally been modelled by assuming optimal canopy functioning, which is not supported by experiments. During this study, we examined how a set of empirical relationships with measurable parameters could be used instead to predict photosynthesis at the leaf and whole-canopy levels. The distribution of leaf N per unit area (N_a_) within the canopy was related to leaf light irradiance and to the nitrogen nutrition index (NNI), a whole-plant variable accounting for plant N status. N_a_ was then used to determine the photosynthetic parameters of a leaf gas exchange model. The model was assessed on alfalfa canopies under contrasting N nutrition and with N_2_-fixing and non-fixing plants. Three experiments were carried out to parameterize the relationships between N_a_, leaf irradiance, NNI and photosynthetic parameters. An additional independent data set was used for model evaluation. The N distribution model showed that it was able to predict leaf N on the set of leaves tested. The N_a_ at the top of the canopy appeared to be related linearly to the NNI, whereas the coefficient accounting for N allocation remained constant. Photosynthetic parameters were related linearly to N_a_ irrespective of N nutrition and the N acquisition mode. Daily patterns of gas exchange were simulated accurately at the leaf scale. When integrated at the whole-canopy scale, the model predicted that raising N availability above an NNI of 1 did not result in increased net photosynthesis. Overall, the model proposed offered a solution for a dynamic coupling of leaf photosynthesis and canopy N distribution without requiring any optimal functioning hypothesis.

## Abstract (precis)

Modelling the distribution of leaf nitrogen is central to specify photosynthetic parameters and simulate canopy photosynthesis using leaf gas exchange models. Nitrogen distribution depends both on soil nitrogen availability and within-canopy light distribution. We propose the use of a set of empirical relationships with measurable parameters to dynamically account for it. The model was assessed on alfalfa canopies under contrasting N nutrition levels. It proved able to predict nitrogen distribution and daily patterns of photosynthesis from leaf to whole canopy for a large range of situations.

## Introduction

A close positive relationship exists between the nitrogen (N) content and photosynthetic capacity of leaves (Field and Mooney 1986; Evans 1989; Hikosaka 2004). Such a relationship is the cornerstone of various approaches proposed to upscale leaf gas exchange models to the whole canopy level (Kull and Jarvis 1995; Leuning 1995; De Pury and Farquhar 1997; Sinoquet *et al*. 2001; Evers *et al*. 2010). Indeed, it has been shown that the within-canopy variability of photosynthetic parameters can be fully specified at a given time by measurements of the spatial distribution of leaf N (Harley *et al*. 1992; Le Roux *et al*. 1999; Braune *et al*. 2009). Changes in leaf N concentration with canopy depth, and the effects of leaf age and leaf light microclimate, have been identified as major sources of spatial variation (Evans 1989). They have been studied extensively in several species and different modelling approaches enable to account for it.

Following the optimization theory, several authors first sought to model N distribution in order to maximize canopy photosynthesis (Charles-Edwards 1981; Field 1983; Hirose and Werger 1987). The outcome was models predicting a leaf N concentration, which paralleled radiation extinction (Kull and Jarvis 1995; Sands 1995). However, there is no *a priori* reason for N distribution to follow such a pattern. On the contrary, empirical observations have consistently indicated that the exponential fall in leaf N with increasing depth into the canopy occurs in most canopies at a slower rate than light extinction (Hirose and Werger 1987; Lemaire *et al*. 1991; Anten *et al*. 1995; Moreau *et al*. 2012). Alternatively, empirical relationships between light extinction and leaf N have been used to mimic a local light acclimation and model leaf N distribution by considering potential departures from the light gradient (Sellers *et al*. 1992; Anten *et al*. 1995; Prieto *et al*. 2012). The approach is usually based on a coefficient of N allocation (*k*_N_) that shapes the N profile with respect to relative light extinction (*I*/*I*_0_), and a reference leaf N concentration at the top of the canopy (N_up_):

(1) $${\text{N}}_{a}={\text{N}}_{\mathrm{up}}{\left(\frac{I}{I_{0}}\right)}^{k_{N}}$$

Leaf N distribution is not solely a function of light and age, however. It is also dependent on mineral N availability (Hikosaka *et al*. 1994; Lötscher *et al*. 2003), N demand to support plant growth and more generally on the N status of plants (i.e. the relative satisfaction of plant N demand, Lemaire and Gastal 1997). The N demand of a plant at any time in its cycle is generally defined as the amount of N necessary to sustain maximum plant growth. Nitrogen demand is tightly related to the standing crop mass. On a mass increment basis, it decreases as biomass increases, resulting in an apparent dilution of plant N concentration with plant growth (Greenwood *et al*. 1990; Gastal *et al*. 2015). Canopy N content and leaf N distribution, thus, respond not only to changes in the fertilization rate (Bélanger *et al*. 1992; Dreccer *et al*. 2000) but also to all factors that affect the plant growth rate (e.g. temperature and CO_2_ concentration; Pettersson and McDonald 1994). Empirical plant N status indices have been developed to account for both aspects and help to diagnose crop N requirements. For instance, the nitrogen nutrition index (NNI) was assessed on plants as different as C3 annual crops (e.g. Justes *et al*. 1994; Colnenne *et al*. 1998), C4 grasses (Plénet and Lemaire 1999) and perennial forage plants (Lemaire *et al*. 1984, 1985). This is based on the concept of critical N dilution that can be applied in dynamic terms and is able to account for temporal changes in the N nutrition of crops (e.g. STICS crop model, Brisson *et al*. 2009).

Modelling the effect of the interaction between light acclimation and N limitations on the distribution of leaf N and photosynthetic characteristics has received comparatively less attention (Thornley 1998). One challenge is that light acclimation is a local process driven by the leaf light microclimate (Evans 1989; Hikosaka *et al*. 1994), whereas N demand, plant N status and N allocation are defined at the whole-plant scale (Givnish 1988; Lemaire and Gastal 1997; Kull 2002; Gastal *et al*. 2015). Empirical relationships between light extinction and leaf N generally refer to static canopies at a given developmental stage, and their parameters need to be adjusted between years, sites or N treatments (Prieto *et al*. 2012). To date, dynamic coupling with plant growth has, thus, mainly been achieved using approaches based on the optimal distribution theory (Johnson *et al*. 2010). Some studies demonstrated a significant relationship between canopy NNI and the *k*_N_ and N_up_ parameters (Lötscher *et al*. 2003; Farruggia *et al*. 2004; Gastal *et al*. 2015). These relationships could be tested to make predictions of photosynthetic parameters under contrasting N availabilities without any *a priori* assumptions regarding optimal functioning of the canopy. Such a model would offer a solution to dynamically simulate the interactions between light and N based on parameters that can be directly measured.

During this study, we developed and assessed a model coupling an empirical canopy N distribution model with a leaf gas exchange model derived from Farquhar *et al*. (1980). The distribution of leaf N content per unit area (N_a_) was related to leaf light irradiance and to the canopy NNI. The objectives were to determine whether such an empirical approach to leaf N distribution could be used to specify spatial and temporal changes in leaf gas exchange under fluctuating light and N availability. Alfalfa was chosen as a model species because its leaf N distribution has already been described extensively under non-limiting N and because this species presents limited age dependency of leaf characteristics (Lemaire *et al*. 1991, 2005; Evans 1993).

## Methods

### Model description

#### Canopy N distribution model

We assumed that spatial and temporal variations in leaf N content per unit area (N_a_) within the canopy and in the course of plant growth can be deduced from leaf light exposure and plant N status. The effect of relative leaf irradiance on relative N_a_ was taken into account using Eq. (1) with the two parameters N_up_ and *k*_N_. The effect of N limitation was assumed to affect whole-canopy N content in leaves by modulating these two parameters. The NNI was considered to account for the effect of canopy N status (integrating the effects of soil mineral N and nodule fixation on internal N availability). At a given time, NNI was defined as:

(2) $$\text{NNI}=\frac{{\text{N}}_{m}}{{\text{N}}_{c}}$$

where N_m_ represents the actual plant N concentration and N_c_ the critical plant N concentration (g N 100 g^−1^ plant) corresponding to its mass *W* (given by equation N_c_ = 4.8*W*^−0.33^ in alfalfa, Lemaire *et al*. 1985). When NNI is close to 1, the plant N status is considered as near optimum. Departures from 1 indicate deficiency (NNI < 1; the intensity of deficiency is then equal to 1 − NNI) or excess N (NNI > 1, the intensity of excess is then equal to NNI − 1). Following Farruggia *et al*. (2004), a linear response of N_up_ to NNI was considered:

(3) $${\text{N}}_{\mathrm{up}}={\text{N}}_{\mathrm{up}}^{\mathrm{opt}}+a_{2}(\text{NNI}-1)$$

where ${\text{N}}_{\mathrm{up}}^{\mathrm{opt}}$ represents the N content of leaves exposed to incoming photosynthetically active radiation for a NNI of 1 and *a*_2_ represents the dependency of upper leaf N content on plant N status. Similarly, the coefficient of N distribution relative to the light gradient was assumed to depend on NNI:

(4) $$k_{N}=k_{N}^{\mathrm{opt}}+a_{3}(\text{NNI}-1)$$

where $k_{N}^{\mathrm{opt}}$ represents the allocation coefficient for a NNI of 1 and *a*_3_ represents the dependency of this coefficient on plant N status.

#### Leaf gas exchange model

The leaf gas exchange model is described in details in Prieto *et al*. (2012) and has originally been assessed on grapevine. It combines the biochemical photosynthetic model developed by Farquhar *et al*. (1980) with a semi-empirical stomatal conductance model that was originally proposed by Ball *et al*. (1987) and then modified by Leuning (1995). All the equations, variables and parameters are presented in Tables A1–A3. The coupling of this leaf gas exchange model with the previously presented canopy N distribution model was performed through the dependency of the principal photosynthesis parameters (value of *V*_cmax_, *J*_max_, triose phosphate utilization rate (TPU) and *R*_d_ at 25 °C) to N_a_. A linear relationship was assumed [Eq. (A9)] (Harley *et al*. 1992; Le Roux *et al*. 1999; Braune *et al*. 2009).

### Model calibration

Three experiments were carried out at the INRA Lusignan research station, France (46.43N, 0.18E), to calibrate this model and assess the impacts of light, N nutrition and leaf age on the distribution of leaf photosynthetic parameters in alfalfa (*Medicago sativa*). The three experiments were based on the same cultivars (cv. ‘Orca’ as a regular N fixing material, and cv. ‘Agate NF’ as a non-N_2_-fixing material, Barnes *et al*. 1990).

#### Experiment 1

The first experiment was performed in a growth chamber between March and June 2010. All plants were grown in 1.5-L pots (10 × 20 cm cylindrical pots) filled with an N-free substrate (fine quartz sand, 0.8–1.4 mm mesh). The pots were arranged in a quincunx and two plants were transplanted into each pot, resulting in a planting density of ∼230 plants m^−2^. Three canopies comprising 81 pots each (i.e. 162 plants each) were grown under contrasting N availabilities at 22 °C/17 °C (day/night) under a 14-h photoperiod. The incident photosynthetic photon flux density (PPFD) was ∼400 µmol m^−2^ s^−1^. Each canopy was surrounded by a row of border plants grown under the same conditions. Two of these canopies were sown using the ‘Orca’ cultivar and were ferti-irrigated every 4 h (daily amount of 200 mL pot^−1^) with either a complete nutrient solution (N+, 8 mmol N) or a low N nutrient solution (N−, 0.5 mmol N). The N concentration of the N+ solution was non-limiting for growth and prevented the nodulation of alfalfa roots. With the N− solution however, nodulation and N fixation did occur in the Orca cultivar (with natural strains of rhizobium, since the plants were not inoculated). The third canopy was sown with the ‘Agate NF’ cultivar grown with the N− nutrient solution, so that N fixation could not compensate for low mineral N availability. In order to induce a size hierarchy into the canopy, and to decorrelate the vertical position of leaves from their age, alternate rows were sown with a 17-day delay in each canopy. The study focussed on the initial growth period (no defoliation). Two samplings were performed in order to characterize the leaf N distribution. The plants were at the 12th visible leaves stage (40 days after the first sowing) and beginning of bloom stage (58 days after the first sowing), based on development of the Orca-N+ canopy. At each date, eight pots (16 plants) were collected from the centre of each canopy.

#### Experiment 2

The second experiment was performed outdoors between April and August 2009 using the ‘Orca’ cultivar. The average incident PPFD was ∼725 µmol m^−2^ s^−1^. All plants were grown in individual 1-L pots (5 × 52 cm cylindrical pots), resulting in a plant density of 460 plants m^−2^. The canopy was made up of 100 study pots surrounded by 3 rows of border plants grown under the same conditions. All pots were filled with a growing medium that comprised sterile potting mix sand and clay-sandy-loam soil from a field in Lusignan (1 : 1 : 1, v/v). They were ferti-irrigated three times a day with the N+ nutrient solution. At the end of the second regrowth (beginning of bloom stage), 20 plants were sampled from the centre of the canopy for the characterization of leaf N distribution. The plants in this canopy had previously been shown to be highly size structured (Baldissera *et al*. 2014).

#### Experiment 3

The third experiment was carried out in a greenhouse between February and June 2012 using the ‘Orca’ cultivar. The average incident PPFD was ∼540 µmol m^−2^ s^−1^. All plants were grown in 1.1-L pots (10 × 10 × 11 cm) filled with an N-free substrate (fine quartz sand, 0.8–1.4 mm mesh). A single plant was transplanted into each pot, resulting in a density of 100 plants m^−2^. Just after transplantation, the seedlings were inoculated with a solid commercial preparation for the coating of alfalfa seeds (*Sinorhizobium meliloti*, strain 2011, Becker Underwood). The pots were automatically ferti-irrigated five times a day with a complete nutrient solution devoid of mineral N (N0, 0 mmol N). The nutrient solution was sampled weekly to determine the absence of $\text{N}{{\text{O}}_{3}}^{-}$ and $\text{N}{{\text{H}}_{4}}^{+}$ and ensure that N fixation was the only source of N supplied to the alfalfa plants. The experimental design consisted of 4 contiguous blocks of 49 pots each. At the end of the initial growth period (mid-bloom stage), four plants were sampled from the centre of each block in order to characterize the leaf N distribution.

#### Measurement of canopy N distribution and NNI

At each sampling date specified in the three experiments, each plant was separated into stems, flowers (when present) and leaves. The leaves were subdivided into 10 cm strata from the bottom to the top of the plant. The leaf area into each strata was determined using an LI-3100 planimeter (LI-COR, Lincoln, NE, USA). Plant samples were dried at 60 °C for 2 days, weighed to determine the dry mass and finally ground in a vibrating ball mill (MM400, Retsch GmbH and Co., Haan, Germany). Each sample was analysed with an elemental analyser to determine the N content (model EA 1108, Carlo Erba Instruments, Milan, Italy). For each canopy, the leaf area index (LAI) was calculated as the average leaf area of the collected plant multiplied by the plant density. The NNI was calculated as the ratio between the canopy N concentration (N_m_) and the critical N concentration (N_c_, Eq. 2).

#### Leaf photosynthetic capacity

In Experiments 1–3, gas exchanges were measured using the same procedure with a portable Licor 6400 photosynthesis system (LI-6400, LI-COR). The photosynthetic parameters were determined through the response of *A* to the internal CO_2_ concentration (*C*_i_) at the sub-stomatal level (*A*–*C*_i_ curves). Different levels of *C*_i_ were obtained by modifying the ambient CO_2_ concentration (*C*_a_) in the leaf measurement chamber. The *A*–*C*_i_ curves were determined as proposed by Long and Bernacchi (2003). Each *C*_a_ step was maintained for 5 min in order to record stable values. The three parameters (*V*_cmax_, *J*_max_ and TPU) were estimated simultaneously by fitting the Farquhar model to the whole *A*–*C*_i_ curve according to the procedure proposed by Sharkey *et al*. (2007). All curves were determined at 1500 µmol m^−2^ s^−1^ of PPFD, while the leaf temperature was controlled at 25 °C and the vapour pressure deficit (VPD) between the leaf and the air was kept at 1 ± 0.5 kPa. Night respiration was estimated at the end of the night on a subsample of leaves that had previously been used for photosynthesis measurements (*A*–*C*_i_ curves). A different leaflet from the same leaf was used. Night respiration was considered to be equal to day respiration (*R*_d_).

In each of the canopies studied, leaves were sampled at three to four levels from the bottom to the top of the canopy, just before the plants were collected to assess canopy N content and leaf N distribution. Measurements were made on the central leaflet of primary leaves. A total of 102 *A*–*C*_i_ curves were analysed over the 3 experiments (70, 20 and 12 for Experiments 1, 2 and 3, respectively).

#### Stomatal conductance

In Experiment 2, the daily evolution of leaf transpiration was recorded on leaves with contrasting positions within the canopy. Measurements were carried out during a series of sunny and cloudy days in summer. The parameters of the stomatal conductance model [Eq. (A10)] were estimated using the data obtained during a period which cumulated ∼5 days of measurements.

#### Leaf traits

For each of the leaves on which *A*–*C*_i_ curves were determined, the leaf age was calculated by the thermal time difference (in °Cd) between the date of measurement and the date of leaf appearance. Thermal time was calculated from the daily integration of air temperatures minus the base temperature (5 °C). Immediately after the gas exchange measurements, the three leaflets were collected and scanned (Konica Minolta C352/C300, Konica Minolta Sensing, Osaka, Japan). The leaf area was determined using image analysis (ImageJ software, http://rsbweb.nih.gov/ij/). The leaves were then dried at 60 °C for 2 days, weighed to determine the dry mass and then ground in a vibrating ball mill (MM400, Retsch GmbH and Co.). Leaf samples were analysed with an elemental analyser (model EA 1108, Carlo Erba Instruments) to determine their N concentration. The specific leaf area (SLA, m^2^ g^−1^), leaf N content per unit dry mass (%) and leaf N content per unit of area (N_a_, g N m^−2^) were then calculated.

#### Determination of local light conditions

In Experiment 1, the PPFD values at the top of the canopy and at the leaf level (for each leaf used for the *A*–*C*_i_ characterizations) were measured using a portable LI-189 quantum meter (LI-COR). In addition, in all the canopies studied, the vertical distributions of leaf area measured at each sampling date were used to compute light extinction and average PPFD levels corresponding to each leaf stratum, using the RATP model (Sinoquet *et al.* 2001). A leaf angle distribution was derived from measurements of alfalfa architecture in Experiment 3 (see Barillot *et al*. 2011 for details).

### Assessment of the leaf gas exchange model at the leaf level

A separate experiment was carried out between March and June 2011 according to the same design as Experiment 1. A data set of 10 leaves was used to assess the ability of the model to predict leaf N from canopy NNI and local leaf irradiance. These leaves were collected from the Orca-N+ and Orca-N− treatments (NNI values of 1.1 and 0.9, respectively) at the beginning of the bloom stage. To evaluate the ability of the model to simulate responses to rapid changes in environmental conditions, the sampled plants were placed outdoors and daily evolutions of the leaf gas exchange were recorded. Measurements were taken during sunny and cloudy days on leaves at different heights within the canopies. On very cloudy days, the plants were placed under a shelter to protect the material from the rain. A total of 14 days were analysed. The incident PPFD, leaf temperature, VPD and *C*_a_ were measured.

### Assessment of the leaf gas exchange model at the whole-canopy level

The behaviour of the leaf gas exchange model when upscaled to the whole-canopy level was also assessed. The leaf N distribution was simulated for contrasting canopies (i.e. LAI values of 1.5, 3 and 5 m^2^ m^−2^) at NNI values ranging from 0.3 to 1.4. Leaf area was assumed to be distributed homogeneously into eight vertical strata, and the leaf N concentration in each stratum was assumed to be acclimated to the relative light irradiance integrated over the day. A leaf angle distribution was derived from measurements of alfalfa architecture in Experiment 3 (see Barillot *et al*. 2011 for details). The light distribution within the canopy was calculated hourly using the RATP model (Sinoquet *et al.* 2001). Simulations were performed for contrasting days in the series used for validation at the leaf level. Net photosynthesis was calculated within each stratum and then summed to determine aboveground whole-canopy net gas exchanges.

### Statistical analyses

Statistical analyses were performed using R software (https://www.r-project.org/). Curve fittings were realized with the nls procedure for Eq. 2 and with the lm procedure for linear regressions (Eqs 3 and 4). Analyses of covariance (ANCOVAs, lm procedure) were used to test for the effects of continuous and categorical variables simultaneously and to compare the slopes and intercepts of linear relationships between N concentration and photosynthetic parameters.

Predicted and measured values of leaf N concentration and net photosynthesis were compared using the root mean square error (RMSE) and bias (Bias) of the model, calculated as follows:

(5) $$\text{RMSE}=\sqrt{\frac{\sum_{i=1}^{n}{\left(s_{i}-m_{i}\right)}^{2}}{n}}$$

(6) $$\text{Bias}=\frac{\sum_{i=1}^{n}\left(s_{i}-m_{i}\right)}{n}$$

where *s_i_* and *m_i_* are the *i*th simulated and measured values, respectively, and *n* is the number of observations.

## Results

### Impact of NNI on the relationship between leaf irradiance and leaf N

**Figure 1.**
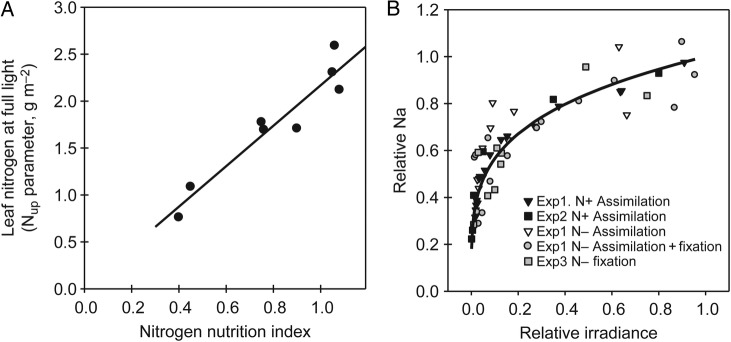
Relationships between (A) canopy NNI and leaf N concentration at the top of the canopy (N_up_ = 2.15 × NNI + 0.02, *r*^2^ = 0.91), and (B) relative leaf irradiance and leaf N concentration relative to the leaf N concentration at the top of the canopy (*k*_N_ = 0.247; *r*^2^ = 0.73).

The relationship between N_a_ and relative leaf irradiance was markedly affected by the N nutrition of the plants. Table 1 summarizes the parameters obtained by fitting Eq. 1 to the different N nutrition situations studied. Parameter N_up_ was the most affected, ranging from ∼2.4 g m^−2^ under N+ treatments to 0.8 g m^−2^ in N− plants reliant on mineral N assimilation alone. It related linearly to the NNI of the plant stand (Fig. 1A; Eq. 3). Variations in N_up_, thus, reflected variations in N nutrition and internal N availability. By comparison, parameter *k*_N_, which accounted for N allocation with respect to relative leaf irradiance, displayed little variation. For *k*_N_ = 1, the N gradient parallels the light gradient within the canopy. All observed values were clearly inferior to unity (<0.5), indicating a more-than-proportional N allocation to leaves with high irradiance and making the N concentration decrease more slowly than relative irradiance. Most *k*_N_ values were within the narrow range between 0.2 and 0.3 (except for one 0.09) with no clear relation to NNI. A single *k*_N_ parameter (0.25) enabled us to fit the normalized N_a_ distributions (Fig. 1B).

**Table 1.** Canopy characteristics and N distribution parameters determined during the different experiments and N treatments studied. Parameters were obtained by fitting Eq. 1 to the N content measured in leaves separated in 10-cm strata. Standard errors are indicated in brackets.

Experiment	Nutrient solution	N acquisition mode	LAI	N_up_	*k*_N_	*r*^2^
1	N+	Assimilation	5.1	2.12 (0.075)	0.23 (0.013)	0.95
1	N+	Assimilation	2.6	2.31 (0.106)	0.24 (0.020)	0.96
2	N+	Assimilation	8.1	2.59 (0.123)	0.21 (0.021)	0.96
1	N−	Assimilation	1	1.09 (0.259)	0.20 (0.088)	0.64
1	N−	Assimilation	0.7	0.76 (0.130)	0.09 (0.312)	0.20
1	N−	Assimilation + fixation	3.3	1.77 (0.134)	0.15 (0.033)	0.87
1	N−	Assimilation + fixation	2.1	1.69 (0.072)	0.29 (0.031)	0.94
3	N0	Fixation	2.0	1.71 (0.181)	0.24 (0.054)	0.75

The effect of leaf age on N_a_ distributions was also assessed [**see Supporting Information—Figs S1 and S2**]. Due to an upward age gradient in alfalfa canopies, leaf nitrogen per unit area was related to both leaf age and local irradiance when considered separately. A multiple regression analysis confirmed the dominant effect of relative irradiance (*t*-value = 5.11; *P* < 10^−6^), but showed a non-significant impact of leaf age *per se* and no interaction with irradiance (*t*-value = 1.54; *P* > 0.12 for the age term).

The canopy N distribution model was parameterized on the basis of these relationships (*a*_2_ = 2.15, ${\text{N}}_{\mathrm{up}}^{\mathrm{opt}}=2.17,$ *a*_3_ = 0 and $k_{N}^{\mathrm{opt}}=0.25$). **Supporting Information—Figure S3** shows the change in leaf N concentration as a function of relative leaf irradiance and NNI as predicted by this model.

### Parameters of the photosynthetic and stomatal conductance model

**Figure 2.**
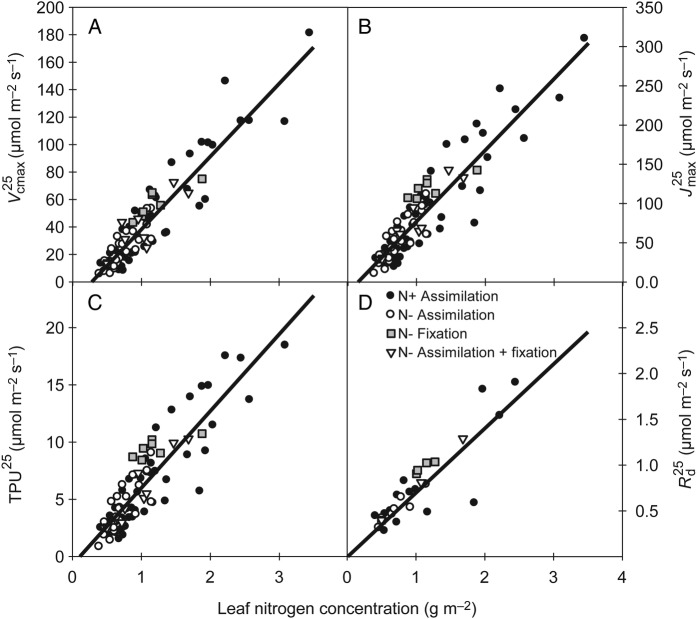
Relationship between the values of photosynthetic parameters at a leaf temperature of 25 °C and leaf N concentration (N_a_) across the different experiments and N treatments studied. Linear relationships were found for $V_{\mathrm{cmax}}^{25}$ (A, $V_{\mathrm{cmax}}^{25}=53.03{\text{N}}_{a}-14.74$, *r*^2^ = 0.86), $J_{\max}^{25}$ (B, $J_{\max}^{25}=90.91{\text{N}}_{a}-13.83$, *r*^2^ = 0.83), TPU^25^ (C, TPU^25^ = 6.72N_a_ − 0.72, *r*^2^ = 0.78) and $R_{d}^{25}$ (D, $R_{d}^{25}=0.69{\text{N}}_{a}-0.005$, *r*^2^ = 0.77), respectively.

Parameters $V_{\mathrm{cmax}}^{25},$ $J_{\max}^{25},$ TPU^25^ and $R_{d}^{25}$ were related linearly to N_a_ (Fig. 2). The range of values observed for the different leaf parameters varied significantly between experiments, in relation to the minimum and maximum values taken by N_a_ (i.e. up to 2.8, 2, 1.8 and 1.1 g N m^−2^ in canopies relying on the N+ solution, N− solution and fixation, and fixation only and N− solution only, respectively). The range of variations in N_a_ values resulted from both the N nutrition of plants and leaf-to-leaf variations in the light microclimate. However, a single relationship was found for each parameter between *P*^25^ and N_a_, independently of the N nutrition. No significant difference in the slopes (ANCOVA, *t*-value <0.91; *P* > 0.38 for interaction terms between *P*^25^ and N_a_) and intercepts (ANCOVA, *t*-value <0.31, *P* > 0.75) were found between the N treatments. A larger dispersion of the points within the ‘N+-assimilation’ data set was observed. This was due to a difference between indoor and outdoor N_a_ values as shown by slightly higher intercepts in Experiment 2 (ANCOVA, *t*-value < −3.84, *P* < 0.001 for the intercept term).

An unique set of measurements from Experiment 2 was used to determine the stomatal conductance parameters (*a*_1_, *D*_o_). It contained leaves from different positions within the canopy and days with contrasting meteorological conditions. Parameter values are presented in Table A2.

### Quantitative assessment of the N distribution model

**Figure 3.**
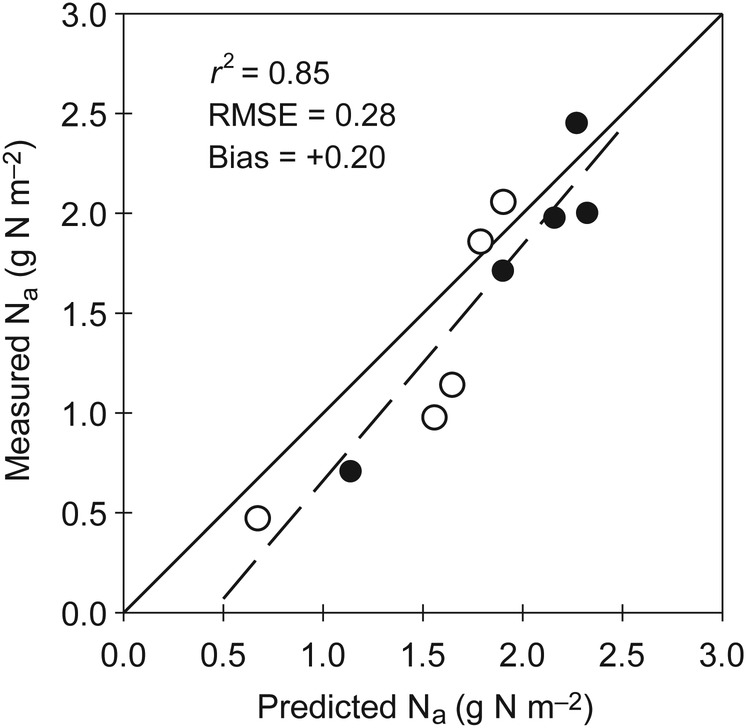
Relationship between leaf N concentrations (N_a_) observed at various positions within the canopy and the corresponding values simulated. Open and filled symbols indicate canopies grown with N− and N+ nutrient solutions, respectively.

Figure 3 compares the simulated N_a_ values (Eqs 1 and 3) with values measured on leaves at various heights within canopies grown under low and high N availability (NNI ranging from 0.45 to 1.1). Most inter-leaf variance in N_a_ values was explained by the N distribution model (*r*^2^ = 0.85). The model error remained low (RMSE = 0.28 g N m^−2^), but a significant positive bias was observed. Predicted values of N_a_ appeared to be slightly higher on average (Bias = +0.20 g N m^−2^), particularly in leaves at an intermediate height within the canopy.

### Quantitative assessment of the leaf gas exchange model

**Figure 4.**
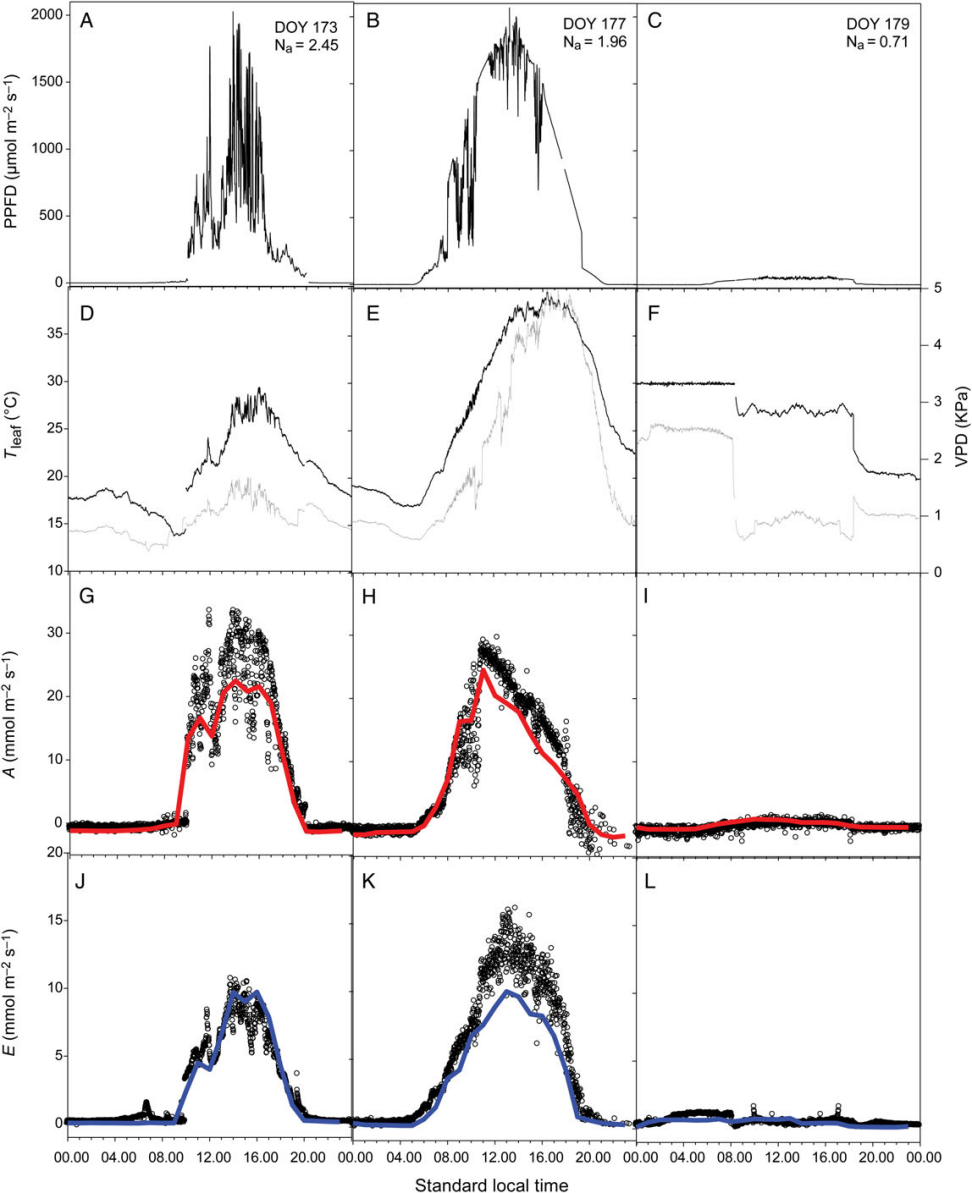
Measured instantaneous PPFD at the leaf level (A–C), leaf temperature (black) and VPD (grey, D–F), and the measured (open circles) and predicted (solid line) net photosynthesis (G–I), and transpiration rates (J–L), for three leaves in Lusignan in 2011. DOY, day of the year.

**Figure 5.**
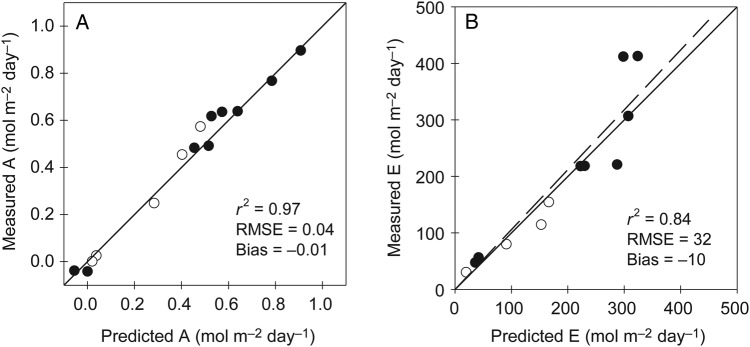
Comparison of measured and predicted values of daily net photosynthesis (A) and transpiration rates (B). Dashed lines: regressions between measured and predicted values; solid lines: 1 : 1 relationships. Open and filled symbols indicate leaves from canopies grown with N− and N+ nutrient solutions, respectively.

The photosynthesis and transpiration sub-models were further assessed using directly measured leaf N_a_. In a first step, the photosynthetic parameters $\left(V_{\mathrm{cmax}}^{25},\;J_{\max}^{25},TPU^{25}\;\text{and}\;R_{d}^{25}\right)$ were calculated using measured N_a_ as an input. The gas exchange model was then run to simulate the daily patterns of *A* and *E* in a range of contrasting leaves (taken from various heights within canopies grown under low and high N availability). The model correctly predicted the diurnal patterns of *A* and *E* in various leaves under contrasting environmental conditions (Fig. 4). Cumulated over a day, the relationship between the observed and simulated values of *A* and *E* did not differ significantly from the 1 : 1 line (Fig. 5; *P* < 0.05). The model accurately estimated the diurnal patterns of *A* and its variation associated with climatic scenarios and leaves under high or low N status (RMSE = 0.04, no significant bias). The predictions also agreed satisfactorily for *E*, but the model errors were greater. Significant discrepancies were observed on *E* predictions for leaves with a high N content on sunny days (e.g. day of the year (DOY) 177 for a leaf at 1.96 g N m^−2^, Fig. 4H). An underestimation of transpiration of up to 20 % was observed under such conditions. This bias did not result from unpaired temporal predictions at a particular time of the day, but from a general underestimation throughout the day.

### Model predictions of the whole-canopy response to N availability

**Figure 6.**
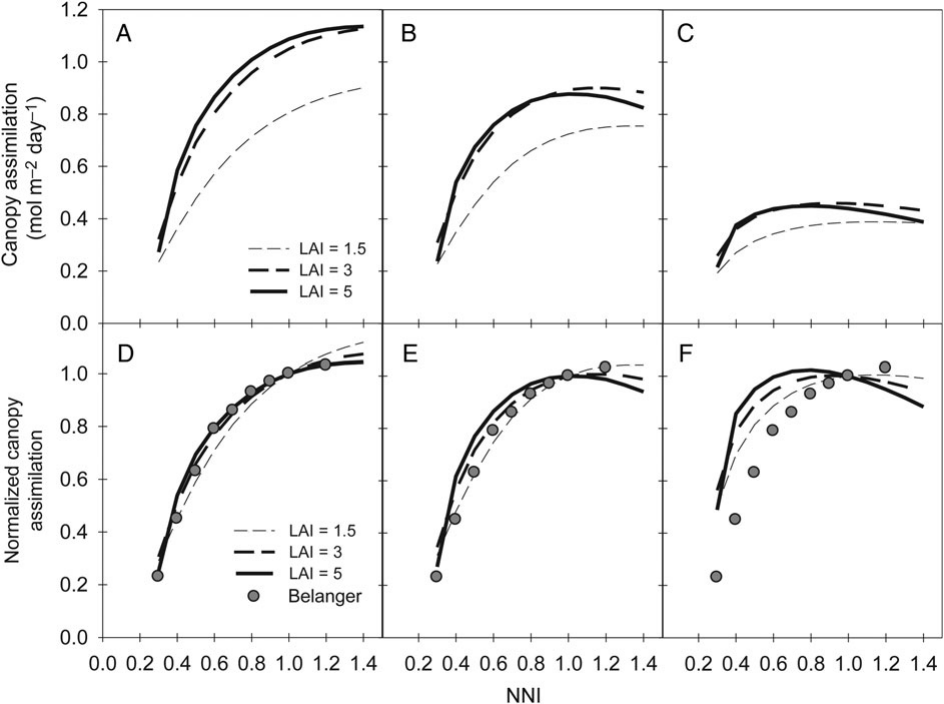
Simulations for three contrasting days of whole-canopy net assimilation in response to changes in the canopy NNI and LAI (A–C) and their corresponding responses normalized by the assimilation rate at an NNI value of 1 (D–F). Grey circles in the lower panels represent the relative reduction in radiation use efficiency measured by Bélanger *et al*. (1992) in response to NNI.

The behaviour of the model integrated at the whole-canopy level was assessed for canopies growing under a range of N availabilities. Examples of daily integrated canopy assimilation are presented in Fig. 6 for three contrasting days (DOY 176, 177 and 157 with an average PPFD decreasing from 709 to 610 and to 263 µmol m^−2^ s^−1^ and average air temperatures of 20.1, 26.3 and 17.9 °C, respectively). All canopies presented a saturating response curve to N availability. As expected, canopy assimilation was lower during cloudy days (Fig. 6A–C). Canopies with a LAI lower than that required for canopy closure (LAI below 3 m^2^ m^−2^) always displayed a lower assimilation rate per unit of soil area. Further increasing the LAI after canopy closure (LAI above 3 m^2^ m^−2^) did not improve canopy assimilation. The threshold at which canopy assimilation ceased to respond to N availability was very close to an NNI value of 1 for closed canopies during sunny and moderately cloudy days (Fig. 6D and E) and for open canopies during very cloudy days (Fig. 6F). Slight shifts of threshold were predicted, depending on the canopy LAI and light availability. Open canopies appeared to be more able to valorize high N availability and displayed delayed thresholds (e.g. at an NNI of ∼1.2 on sunny days). In contrast, dense canopies presented anticipated thresholds that were particularly apparent on cloudy days.

## Discussion

### A simple empirical model to link leaf N distribution with plant N status and light distribution

To date, modelling the interaction between N limitations and light acclimation has been tackled using ‘goal seeking’ or optimal distribution theory (Chen *et al*. 1993; Thornley 1998; Johnson *et al*. 2010). Our study demonstrated how a combination of empirical relationships might be a promising option for this purpose too. The strategy proposed is based on modulation of the N_up_ and *k*_N_ parameters used in the empirical distribution model as a function of plant N status (NNI). A linear relationship was found between N_up_ and NNI over the range of alfalfa canopies studied. Similar results had previously been reported in different grass species, where the relationship was shown to be stable under contrasting growth conditions and canopy structures (Farruggia *et al*. 2004; Gastal *et al*. 2015). In these species, N_up_ has even been used as a routine proxy to facilitate the determination of NNI in the field (Louarn *et al*. 2015; Maamouri *et al*. 2015). The second parameter in the empirical relationship, *k*_N_, was shown to be independent of NNI during the present study. Depending on the species, however, contradictory results have been reported concerning the effect of N limitation on *k*_N_. In some cases, limited effects have been observed (Sinclair and Shiraiwa 1993; Sadras *et al*. 2012), whereas in others, a steeper N gradient has been found in N-stressed plants (Dreccer *et al.* 2000; Milroy *et al*. 2001). Moreau *et al*. (2012) suggested that the size of the canopy (indirectly reduced by N stress), rather than a direct NNI effect, might explain the steeper gradient in N-limited wheat canopies. In line with our results, Lemaire *et al*. (1991) did not show any variation of *k*_N_ in alfalfa canopies at contrasting developmental stages. Different types of plant architecture may affect N reallocation strategies and contribute to explaining these differences in the *k*_N_ response. Some species, such as alfalfa or sunflower (Archontoulis *et al*. 2011), are made up of leaves distributed in different strata along the vertical light gradient, and may adjust more efficiently than long-leaf species (such as grasses or cereals) in which each leaf may simultaneously experience light conditions from the bottom to the top of the canopy. In those cases, the parameter *a*_3_ representing the dependency of *k*_N_ on plant N status (Eq. 3) is likely to take values different from zero.

### Assumptions and potential limitations of the leaf N distribution model

Species differ in the plasticity of their leaf traits and in the within-canopy variation of photosynthetic characteristics (Niinemets *et al*. 2015). The present model assumes that the distribution of leaf N is mainly driven by two factors: the light gradient within the canopy and the plant N status. No significant age effects were recorded in alfalfa, as previously shown in several other species (Evans 1993; Hikosaka *et al*. 1994). This is not a general feature however, and many plant species display age-dependent leaf traits, such as decreasing SLA in ageing leaves for instance. This can alter the light–N_a_ relationship and limit the validity of our model (Prieto *et al*. 2012). In their recent review, Niinemets *et al*. (2015) distinguished two main groups of species: a first group with high rates of canopy development and leaf turnover, exhibiting highly dynamic light environments, active change photosynthetic characteristics by N reallocation among leaves, and a second group made up of species with slow leaf turnover exhibiting a passive N_a_ acclimation response, primarily determined by the acclimation of leaf structure. The proposed model appears clearly best suited to the first group of species because they are less susceptible to leaf ageing effects.

Another limitation of empirical models is their validity out of their domain of calibration. Other environmental factors, such as water stress (Errecart *et al*. 2014), extreme temperatures or extreme light environments (as shown by the indoor/outdoor effect in our data set), can affect leaf growth and leaf traits. In some cases, this is likely to imply a reassessment model parameter values. Finally, the two-parameter model used [Eq. 1, **see Supporting Information—Table S1**] could present a lack of flexibility in some species. This formalism was previously used on other crops (Moreau *et al*. 2012; Sadras *et al*. 2012), but studies comparing a large number of species have generally relied on three-parameter models because they presented an overall better fit (Lötscher *et al*. 2003; Niinemets *et al*. 2015).

### The N acquisition mode did not affect the photosynthetic parameters

Our study confirmed in alfalfa a linear relationship between Farquhar photosynthetic parameters (*P*^25^) and leaf N per unit leaf area (Field 1983; Evans 1989) and showed that it holds true for leaves in different canopies grown under contrasting mineral N availabilities. Acclimation to light and plant N status both affected the N concentration of leaves, but the N_a_–*P*^25^ relationships remained unchanged, as shown previously (Braune *et al*. 2009). In addition, our study examined the effects of the N acquisition mode in legumes, comparing fixing and non-fixing genotypes of alfalfa under different N nutrition statuses. There was no significant impact of the N acquisition mode on the N_a_–*P*^25^ relationships. We thus showed that, contrary to the whole-plant level (Gosse *et al*. 1986), no extra cost to carbon acquisition was associated with N fixation at the leaf level (Boller and Heichel 1984). No gain, associated with an extra carbon sink, was observed either. Overall, the N_a_–*P*^25^ relationships established during this study on a perennial forage legume displayed slopes (e.g. *S_Na_* of the ${\text{N}}_{a}-V_{\mathrm{cmax}}^{25}$ relationship at 53 µmol g N^−1^ s^−1^) that were intermediate between those of cereals (60 and 63 µmol g N^−1^ s^−1^ for wheat and barley, respectively; Müller *et al*. 2005; Braune *et al*. 2009) and those of C3 trees and vines (e.g. ∼30 and 38 µmol g N^−1^ s^−1^ in walnut tree and grapevine, respectively; Le Roux *et al*. 1999; Prieto *et al*. 2012). This positioning was consistent with other productive grassland species (e.g. 36–50 µmol g N^−1^ s^−1^ reported for cocksfoot and red clover; Wohlfahrt *et al*. 1998).

### Performance of the leaf gas exchange model at the leaf and whole-canopy levels

The gas exchange model correctly estimated daily cumulated values of net assimilation (*A*) and transpiration (*E*) at the leaf level and their diurnal patterns. Model errors, however, were greater with respect to transpiration. This might partly be related to the absence of a direct relationship between the leaf N and stomatal conductance parameters considered in the model. Indeed, the scaling parameter *a*_1_ has been shown to increase for leaves with a very low N concentration (Braune *et al*. 2009). In our case, however, discrepancies in *E* mainly concerned the top leaves under high N availability. An alternative explanation might be a less robust parameterization of the stomatal conductance model. Leaves from a single experiment were used, covering a more limited range of environmental conditions than that encountered in the validation data set (in terms of VPD in particular). Future work will incorporate the response to water deficit in the model, which should enable the refinement of this parameterization.

When upscaled at the whole-canopy level, the gas exchange model coupled with the N distribution model displayed interesting properties regarding the response to N availability. The relationship between N and the assimilation rate switched from a linear function at the leaf level to a saturating function at the whole-canopy scale. Above a certain threshold, the model predicted that an increase in N did not result in increased canopy assimilation. Such a point had previously been reported during numerous experiments comparing a broad range of N fertilization rates (Justes *et al*. 1994; Lemaire and Gastal 1997), and it defines the critical N concentration on which NNI calculations are based. Remarkably, such behaviour emerged from our canopy-integrated model. Furthermore, the NNI value corresponding to this transition happened to be very close to 1 during sunny and moderately cloudy days. This complied with the theoretical definition of NNI, which states that a value of 1 corresponds to the critical N concentration. The threshold was predicted to be lower during cloudy days. However, as in practice, the critical N concentrations are determined from cumulated values of biomass production, and critical N is likely to be primarily driven by sunny days (accounting for most biomass accumulation) rather than cloudy days.

No direct measurements were carried out to quantitatively assess the gas exchange model on alfalfa canopies. However, the simulated plateau values were consistent with previous studies measuring the daily net carbon exchange in closed canopies of alfalfa under non-limiting N. For instance, Heichel *et al*. (1988) reported net rates of 1.17, 0.81 and 0.45 mol CO_2_ m^−2^ day^−1^ in a 2.7 LAI canopy during days with average PPFD values of 1100, 700 and 400 µmol m^−2^ s^−1^, respectively (corresponding roughly to the light conditions prevailing during the 3 days shown in Fig. 6). Similarly, Woodward and Sheehy (1979) reported rates ranging from 0.27 to 1.39 mol CO_2_ m^−2^ day^−1^ after canopy closure during a spring regrowth. As in these two studies, the model outputs concerned the net aboveground carbon exchange of N fertilized alfalfa (with presumably limited N fixation). Allocation to the root system and to the respiration of roots and nodules needs to be implemented in future versions of the model in order to account for a potential cost of N fixation in terms of the carbon balance at the canopy level (Gosse *et al*. 1986). Root growth and respiration indeed represent a substantial share of carbohydrate use in fixing alfalfa (Thomas and Hill 1937; Layzell *et al*. 1981).

Fewer references were available to compare the response induced by N limitation. We, thus, compared the simulation results with those relative to regular non-fixing C3 plants and found a relative reduction in the net assimilation rate measured by Bèlanger *et al*. (1992) in a range of tall fescue canopies (Fig. 6). The simulated response curve of dense canopies on sunny and moderately cloudy days appeared to be particularly close to the measured curve, suggesting a good ability of the upscaled leaf model to capture the N stress response of a whole canopy.

## Conclusions

Overall, the set of empirical relationships introduced in this article to distribute leaf N was simple and effective at predicting leaf N concentration in response to light and plant N status. The leaf gas exchange model proved accurate and produced consistent predictions in terms of whole-canopy assimilation under contrasting soil N availability scenarios. Even if the genericity of the coupled model still needs to be challenged in a broader range of species, this work constitutes a further step towards models that can bridge local acclimation to light with N acquisition and global N nutrition status, without presuming an optimal carbon gain or N distribution. Such a model relies on parameters that can all be directly measured and may help us to infer and better understand the differences in N use efficiency observed between species or genotypes.

## Sources of Funding

This study received support from the Regional Council for Poitou-Charentes (http://www.poitou-charentes.fr) and INRA's Environment and Agronomy Division (INNI and TransfertN projects).

## Contributions by the Authors

G.L., E.L. and J.P. contributed to model development. G.L. and E.F. designed the experiments and conducted measurements. They performed data analyses with the help of S.Z. All of the authors contributed to writing the manuscript.

## Conflict of Interest Statement

None declared.

## Supporting Information

The following additional information is available in the online version of this article –

**Figure S1**. Impact of leaf ageing and relative leaf irradiance on the specific leaf area and leaf nitrogen concentration per unit area in alfalfa.

**Figure S2**. Relationship between leaf age and the residuals of the fit of Eq. 1 to leaf nitrogen concentration.

**Figure S3**. Variations in leaf nitrogen concentrations predicted for alfalfa as a function of the nitrogen nutrition index of the canopy and the relative leaf irradiance.

**Table S1**. Comparison of two- and three-parameter models to account for distribution of leaf nitrogen concentrations with respect to relative irradiance in alfalfa.

Additional Information

## Appendix

**Table A1.** Equations for the photosynthesis and stomatal conductance models.

Equation	Description	No.
Photosynthesis model		
$A=V_{c}-0.5V_{o}-R_{d}=V_{c}[1-(\Gamma^{*}/C_{i})]-R_{d}$	Net photosynthetic rate (µmol CO_2_ m^−2^ s^−1^)	(A1)
$V_{c}=\text{min}\{A_{c},A_{j},A_{p}\}$	Carboxylation rate (µmol CO_2_ m^−2^ s^−1^)	(A2)
$A_{c}=(V_{\mathrm{cmax}}C_{i})/[C_{i}+K_{c}(1+(O/K_{o}))]$	RUBISCO-limited photosynthetic rate	(A3)
$A_{j}=(JC_{i})/(4C_{i}+\text{8}\Gamma^{*})$	RuBP regeneration-limited photosynthetic rate	(A4)
$A_{p}=(3\text{TPU})/[1-(\Gamma^{*}/C_{i})]$	TPU-limited photosynthetic rate	(A5)
$J=(\alpha \cdot \text{PPFD})/\sqrt{1+[(\alpha^{2}\cdot \text{PPF}{\text{D}}^{2})/J_{\max}^{2}]}$	Electron transport rate, dependence on the radiance level	(A6)
$P={\text{e}}^{\left(c-(\Delta H_{a}/RT_{k})\right)}$	Arrhenius function, temperature dependence for *K*_c_, *K*_o_, Γ* and *R*_d_	(A7)
$P={\text{e}}^{\left(c-(\Delta H_{a}/RT_{k})\right)}/(1+{\text{e}}^{\left(\Delta ST_{k}-(\Delta H_{d}/RT_{k})\right)})$	Arrhenius function, temperature dependence for *V*_cmax_, *J*_max_ and TPU	(A8)
$P^{25}=S_{\mathrm{Na}}\cdot {\text{N}}_{a}-b$	Nitrogen dependence function for *V*_cmax_, *J*_max_, TPU and *R*_d_ at 25 °C	(A9)
Stomatal conductance model		
$g_{s}=g_{o}+(a_{1}A)/([1+(\mathrm{VPD}/D_{o})](C_{s}-\Gamma ))$	Stomatal conductance	(A10)
$C_{s}=C_{a}\text{- }A\cdot (1.37/g_{b})$	CO_2_ partial pressure at the leaf surface	(A11)
$C_{i}=C_{a}\text{- }A\cdot [(\text{1}.6/g_{s})+(1.37/g_{b})]$	*C*_i_ value by coupling *A* and *g*_s_	(A12)

**Table A2.** Symbols, values and units of different parameters, variables and constants used in the photosynthetic and stomatal conductance models. ^1^Values taken from Schultz (2003). ^2^Values taken from Harley *et al*. (1992). ^3^Constant used for measurement in the leaf chamber of the LcPro (ADC Lcpro, BioScientific Ltd, Hoddesdon, Hertfordshire, UK).

Symbol	Value	Unit	Description
Photosynthesis model			
*α*	0.20^1^	µmol CO_2_ µmol photon^−1^	Photochemical efficiency or initial quantum yield
Γ*	–	Pa	Compensation point for CO_2_ in the absence of mitochondrial respiration
*A*	–	µmol CO_2_ m^−2^ s^−1^	Net photosynthetic rate
*A*_c_	–	µmol CO_2_ m^−2^ s^−1^	RUBISCO-limited photosynthetic rate
*A*_j_	–	µmol CO_2_ m^−2^ s^−1^	Electron transport rate-limited photosynthetic rate
*A*_p_	–	µmol CO_2_ m^−2^ s^−1^	Triose phosphate utilization-limited photosynthetic rate
c	–	–	Scaling constant
*C*_a_	–	Pa	Ambient CO_2_ partial pressure
*C*_i_	–	Pa	Intercellular CO_2_ partial pressure
Δ*H*_a_	–	kJ mol^−1^	Enthalpy of activation
Δ*H*_d_	200^2^	kJ mol^−1^	Enthalpy of deactivation
*K*_c_	–	Pa	Michaelis–Menten constant of RUBISCO for CO_2_
*K*_o_	–	kPa	Michaelis–Menten constant of RUBISCO for O_2_
*J*	–	µmol electron m^−2^ s^−1^	Electron transport rate
*J*_max_	–	µmol m^−2^ s^−1^	Maximum electron transport rate
N_a_	–	g m^−2^	Area based N content
N_amin_	–	g m^−2^	Minimum value of N_a_ at which *P*^25^ → 0
*O*	21	kPa	Oxygen partial pressure
*P*^25^	–	µmol m^−2^ s^−1^	Value of *V*_cmax_, *J*_max_, TPU or *R*_d_ at 25 °C
PPFD	–	µmol m^−2^ s^−1^	Photosynthetic photon flux density
*R*	0.00831	kJ mol^−1^ K^−1^	Universal gas constant for perfect gases
*R*_d_	–	µmol m^−2^ s^−1^	Mitochondrial respiration in light
Δ*S*	0.635^2^	kJ mol^−1^	Entropy term
*S*_Na_	–	µmol g^−1^ s^−1^	Slope of the relationship between N_a_ and *V*_cmax_, *J*_max_, TPU or *R*_d_
*T*_leaf_	–	°C	Leaf temperature in degrees Celsius
*T*_k_	–	Kelvin degrees	Leaf temperature in Kelvin
TPU	–	µmol m^−2^ s^−1^	Triose phosphate utilization rate
*V*_c_	–	µmol m^−2^ s^−1^	Carboxylation rate
*V*_o_	–	µmol m^−2^ s^−1^	Oxygenation rate
*V*_cmax_	–	µmol m^−2^ s^−1^	Maximum rate of RUBISCO carboxylation
Stomatal conductance model			
*C*_s_	–	Pa	CO_2_ partial pressure at the leaf surface
*g*_b_	2.357^3^	mol m^−2^ s^−1^	Boundary layer conductance
*g*_s_	–	mmol m^−2^ s^−1^	Stomatal conductance
*g*_o_	0.020	mmol m^−2^ s^−1^	Residual stomatal conductance when *A* → 0
VPD	–	kPa	Water VPD
*D*_o_	2.86	kPa	Empirical factor assessing stomata sensitivity to VPD
*a*_1_	12.5	–	Empirical stomatal conductance factor

**Table A3.** Values of c (scaling constant), enthalpies of activation (Δ*H*_a_) describing the temperature response for parameters of the photosynthesis model. ^1^Values taken from Bernacchi *et al*. (2001). ^2^Values taken from Bernacchi *et al*. (2003). ^3^Values taken from Harley *et al*. (1992).

Parameter	Value at 25 °C	c	Δ*H*_a_ (kJ mol^−1^)
*V*_cmax_	–	26.35^1^	65.33^1^
*J*_max_	–	17.7^2^	43.9^2^
TPU	–	21.46^3^	53.1^3^
*R*_d_	–	18.72^1^	46.39^1^
Γ*	42.75^1^	19.02^1^	37.83^1^
*K*_c_	404.9^1^	38.05^1^	79.43^1^
*K*_o_	278.4^1^	20.30^1^	36.38^1^
