# Supplementary material for: An empirical model that uses light attenuation and plant nitrogen status to predict within-canopy nitrogen distribution and upscale photosynthesis from leaf to whole canopy
Source: AoB Plants. 2015 Oct 3;7:plv116. doi: 10.1093/aobpla/plv116 (PMC4635319; doi:10.1093/aobpla/plv116)
Supplement: Additional Information [file supp_7_plv116_index.html]

An empirical model that uses light attenuation and plant nitrogen status to predict within-canopy N distribution and upscale photosynthesis from leaf to whole canopy — An empirical model that uses light attenuation and plant nitrogen status to predict within-canopy nitrogen distribution and upscale photosynthesis from leaf to whole canopy — Additional Information 

# An empirical model that uses light attenuation and plant nitrogen status to predict within-canopy nitrogen distribution and upscale photosynthesis from leaf to whole canopy

## Additional Information

Additional Information

- Additional Information - Doc file
