# Supplementary material for: An empirical model that uses light attenuation and plant nitrogen status to predict within-canopy nitrogen distribution and upscale photosynthesis from leaf to whole canopy
Source: AoB Plants. 2015 Oct 3;7:plv116. doi: 10.1093/aobpla/plv116 (PMC4635319; doi:10.1093/aobpla/plv116)
Supplement: Additional Information [file supp_plv116_plv116supp.doc]

**Figure Sup. A**: Impact of leaf ageing and relative leaf irradiance on the specific leaf area (a and b, respectively) and leaf nitrogen per unit area (c and d) in alfalfa plants grown under two contrasting N nutrition levels. Measurements were obtained from the individual leaves used for photosynthesis measurements in Exp. 1. Open circles and dotted lines refer to the N- treatment (AgateNF cv.), whereas filled circles and full lines refer to the N+ treatment (Orca cv.).

**Figure Sup. B**: Relationship between leaf age and the residuals of the fit of Eq.1 to *N_a_* distributions. Measurements were obtained from the individual leaves used for photosynthesis measurements in Exp. 1. Open circles and dotted lines refer to the N- treatment (AgateNF cv.), whereas filled circles and full lines refer to the N+ treatment (Orca cv.).

**Figure Sup. C**: Variations in leaf nitrogen concentrations (*N_a_*) predicted as a function of the nitrogen nutrition index (NNI) of the canopy and the relative leaf irradiance within the canopy using Eq. 2 and 3 with *a_2_*=2.15, *N_up_^opt^*=2.17, *a_3_*=0. and *k_N_^Opt^* = 0.25.

**Table Sup. A**: Comparison of two and three parameter models to account for distribution leaf nitrogen concentrations (*N_a_*) with respect to relative irradiance (*I/I_0_*) in alfalfa. AIC: Akaike information criterion, BIC: Bayesian information criterion.

|  | BIC | AIC | Resiuals sum of squares | r^2^ |
| --- | --- | --- | --- | --- |
| 2 parameters, Eq1 | -74.7 | -92.2 | 0.5881 | 0.7872 |
| 3 parameters, Lötsher et al. (2003) | -50.5 | -87.1 | 0.5874 | 0.7875 |
| 3 parameters, Niinemets et al. (2015) | -47.7 | -83.5 | 0.6238 | 0.7743 |
